# Bandwidth tunability of graphene absorption enhancement by hybridization of delocalized surface plasmon polaritons and localized magnetic plasmons

**DOI:** 10.1186/s11671-024-03961-6

**Published:** 2024-01-25

**Authors:** Yifan Wu, Qingmiao Nie, Chaojun Tang, Bo Yan, Fanxin Liu, Mingwei Zhu

**Affiliations:** 1 College of Physics and National Laboratory of Solid State Microstructures, Nanjing University, Nanjing, 210093 China; 2 College of Science, Zhejiang University of Technology, Hangzhou, 310023 China; 3 State Key Laboratory of Millimeter Waves, Southeast University, Nanjing, 210096 China; 4 College of Engineering and Applied Sciences and Jiangsu Key Laboratory of Artificial Functional Materials, Nanjing University, Nanjing, 210093 China

**Keywords:** Graphene absorption, Bandwidth tunability, Plasmon hybridization

## Abstract

The bandwidth-tunable absorption enhancement of monolayer graphene is theoretically studied in the near-infrared wavelengths. The monolayer graphene is placed on the silver substrate surface with a periodic array of one-dimensional slits. Two absorption peaks are found to result from the hybridization of delocalized surface plasmon polaritons and localized magnetic plasmons. The positions of absorption peaks are accurately predicted by a coupling model of double oscillators. The full width at half maximum of absorption peaks is largely tuned from about 1–200 nm by changing the array period of slits. The effect of the slit size on absorption peaks is also investigated in detail. Our work is promising in applications for photoelectric devices.

## Introduction

Graphene has both excellent optical and electrical properties, and so is very promising in applications of many photoelectric devices, for example, photodetector [1, 2]. In graphene-based photoelectric devices, maintaining a high light absorption efficiency of graphene usually plays a very important role [3, 4]. Unfortunately, the well-known fine structure constant determines that the absorption efficiency of graphene is only about 2.3% from visible to near-infrared wavelengths [5, 6], when light is normally incident on and passes through a pure graphene monolayer in air. This very low and universal (wavelength-independent) absorption efficiency of 2.3% is not beneficial in the aspect of photoelectric devices. To improve the graphene absorption as far as possible, a variety of physical methods are proposed recently, such as different types of surface plasmon resonances [7–13], critical coupling of guided mode resonances [14–16], Fabry–Perot resonances [17–19], photonic crystal defect states [20–23], attenuated total reflections [24–27], and so on. Through these physical methods, the electromagnetic fields on the graphene surface are enhanced hugely, and thus the graphene absorption is improved greatly [3].

In recent years, largely tuning the graphene absorption bandwidth has been also gaining a lot of attention for different practical applications [28–32]. Graphene-based photodetectors and photovoltaics require a very broad absorption bandwidth, while light emitters need a relatively narrow absorption bandwidth. On one hand, to obtain the broadband graphene absorption, an effective multiple-resonator approach is usually utilized in some papers [33–37]. One the other hand, some high-Q resonances, such as guided mode resonances and lattice plasmon resonances, can be employed to achieve the narrowband graphene absorption [15, 38–41]. In many designed structures for bandwidth-tunable graphene absorption enhancement, the monolayer graphene is commonly sandwiched between different materials, and correspondingly adds the fabrication difficulty in experiment. At present, it remains a huge challenge to realize the bandwidth-tunable graphene absorption enhancement, by designing relatively easily fabricated structures.

In metal nanostructures, Rabi splitting with an anticrossing behavior can be realized by the strong coupling between surface plasmons and different kinds of quantum emitters, such as semiconductor quantum dots [42], molecules [43], J-aggregates [44], and two-dimensional materials [45]. Very recently, strong coupling is also theoretically reported in judiciously integrated semiconducting single-walled carbon nanotubes with metallic nanoelectrodes [46]. It is revealed that the number increase of carbon nanotubes across the barrier can lead to a substantial enhancement in Rabi splitting. In a recent work, we studied ultra-large Rabi splitting as large as 805 meV, by the broadband strong coupling between whispering gallery mode and plasmon resonance in spherical hyperbolic metamaterial cavity [47].

In this work, we theoretically study a relatively simple structure to realize the bandwidth tunability of graphene absorption enhancement, by directly placing the monolayer graphene on the silver substrate surface with a periodic array of one-dimensional slits. The hybridization of the SPPs propagating on the silver substrate surface and the MPs confined within the slits results into two absorption peaks of monolayer graphene, and the interesting Rabi splitting is also shown. The *FWHM* of absorption peaks can be largely tuned from about 1 nm to 200 nm by changing the array period of the slits, and the practical positions of absorption peaks can be also accurately predicted by a coupling model of double oscillators. In addition, we carefully investigate the effect of the geometry size of the slits on the graphene absorption. Our work may hold some promising applications in photoelectric devices.

## Results and discussion

**Fig. 1:**
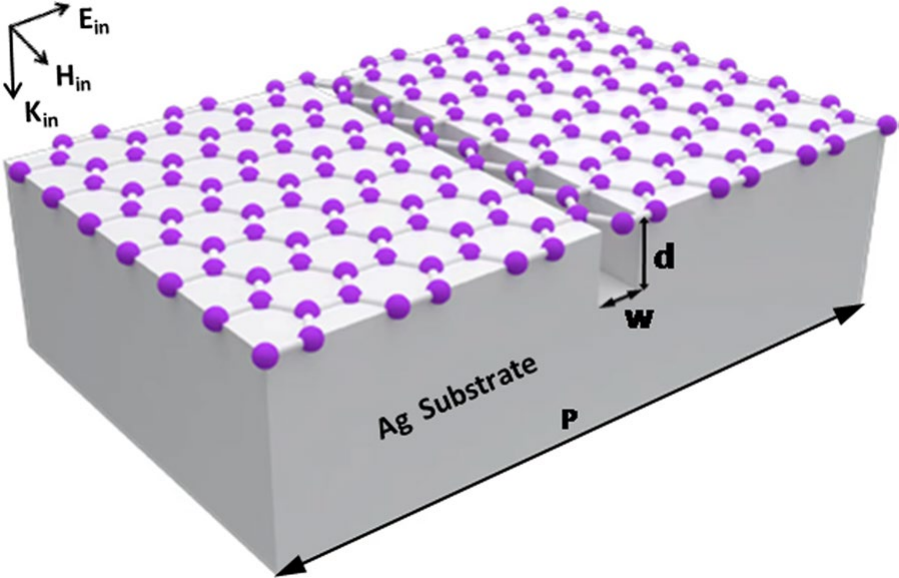
Structure for bandwidth tunability of graphene absorption enhancement by hybridization of delocalized SPPs and localized MPs

In Fig. 1 we show schematically the studied structure that consists of a graphene monolayer on a silver substrate with one-dimensional periodic air slit arrays on its surface. The array period is *p*, and the width and the depth of the slit are *w* and *d*, respectively. As indicated by the arrows, the light is normally incident on the studied structure, with its electric and magnetic fields perpendicular and parallel to the slit, respectively. Under such an incident condition, localized MPs can be excited within the slit [48, 49], and delocalized SPPs can be excited on the surface of the silver substrate. We perform relevant numerical simulations by using the commercial software (https://www.eastfdtd.com). In numerical simulations of electromagnetic waves, the refractive index of silver is from experimental results [50]. The surface conductivity* σ* and the permittivity *ε*_*g*_ of graphene are calculated by the following two analytical expressions [51–55]:

1 $$\sigma = \frac{{ie^{2} k_{B} T}}{{\pi \hbar^{2} (\omega + {i \mathord{\left/ {\vphantom {i \tau }} \right. \kern-0pt} \tau })}}\left( {\frac{{E_{f} }}{{k_{B} T}} + 2\ln (e^{{ - \frac{{E_{f} }}{{k_{B} T}}}} + 1)} \right) + \frac{{ie^{2} }}{4\pi \hbar }\ln \left( {\frac{{2E_{f} - (\omega + {i \mathord{\left/ {\vphantom {i \tau }} \right. \kern-0pt} \tau })\hbar }}{{2E_{f} + (\omega + {i \mathord{\left/ {\vphantom {i \tau }} \right. \kern-0pt} \tau })\hbar }}} \right)$$

2 $$\varepsilon_{g} = \left( {\begin{array}{*{20}c} {1 + {{i\sigma } \mathord{\left/ {\vphantom {{i\sigma } {(\omega \varepsilon_{0} t_{g} )}}} \right. \kern-0pt} {(\omega \varepsilon_{0} t_{g} )}}} & 0 & 0 \\ 0 & {1 + {{i\sigma } \mathord{\left/ {\vphantom {{i\sigma } {(\omega \varepsilon_{0} t_{g} )}}} \right. \kern-0pt} {(\omega \varepsilon_{0} t_{g} )}}} & 0 \\ 0 & 0 & 1 \\ \end{array} } \right)$$

In the expressions, *i* is imaginary unit, *k*_*B*_ is Boltzmann constant, *e* is electron charge, *π* is circular constant, *ω* is angular frequency, *ħ* is reduced Planck constant, and *ε*_*0*_ is vacuum permittivity. The slit arrays can be firstly fabricated by the advanced focused-ion-beam etching technique, and then the prepared graphene monolayer is transferred on the silver substrate [56, 57]. In numerical calculations, a Gauss pulse acts as incident light. Perfectly matched layers are set in the *z*-axis direction, and periodic boundary conditions are set in the *x*- and *y*-axis directions. Inhomogeneous mesh size (*Δs*) with a time step (*Δt* = *Δs*/2*c*) is used: 0.05 nm in graphene, 5 nm in nanoslit, and 20 nm in the other region. Through the above simulation settings, reliable simulation results can be obtained.

**Fig. 2:**
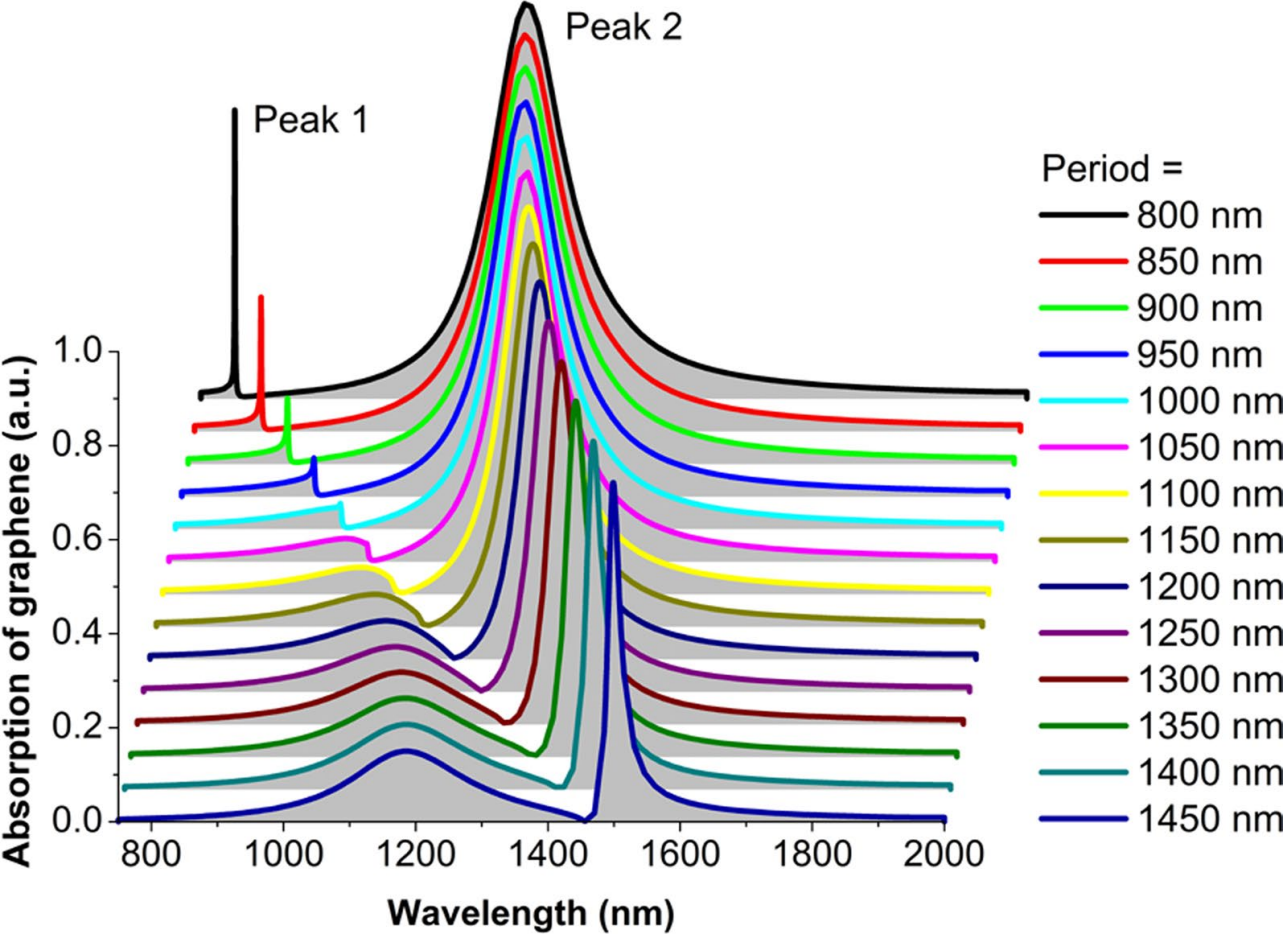
Graphene absorption spectra for different period. Geometric and physical parameters: slit width *w* = 30 nm, slit depth *d* = 150 nm, Fermi energy *E*_*f*_ = 0.30 eV, relaxation time *τ* = 0.50 ps, graphene thickness *t*_*g*_ = 0.34 nm, temperature* T* = 300 K

**Fig. 3:**
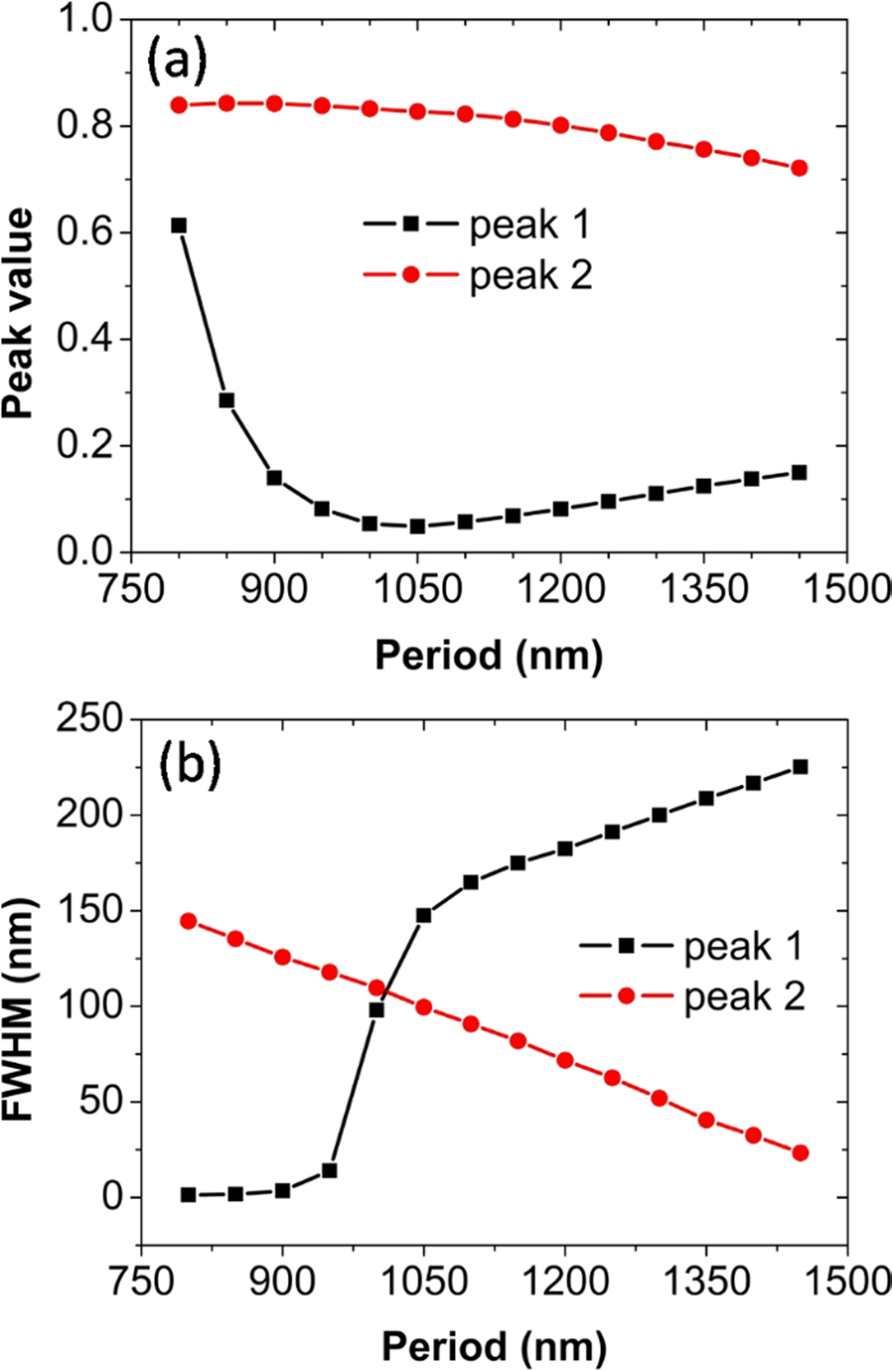
(**a**) Peak value and (**b**) *FWHM* for different period

In Fig. 2 we present normal-incidence graphene absorption spectra from 750 to 2000 nm, for the period *p* to have an increase from 800 to 1450 nm with a step of 50 nm. For each spectral line to be seen clearly, we have vertically and horizontally offset the absorption spectra by 90% and 10%, respectively. For the period *p* is equal to 800 nm, we observe two noticeable graphene absorption peaks. One absorption peak is very narrow and the other absorption peak is relatively broad, which are labeled as peak 1 and peak 2 in Fig. 2, respectively. These two absorption peaks are demonstrated later to result from hybridization of delocalized SPPs and localized MPs. When the period *p* increases, the absorption value and the *FWHM* of these two absorption peaks will have an obvious change. To clearly exhibit the change trends, in Fig. 3 we present the dependence of the peak value and the *FWHM* on the array period *p*. For peak 1, the peak value firstly decreases from 0.60 to 0.05 and then increases to 0.15, and the *FWHM* continuously increases from 1.28 to 225 nm. For peak 2, the peak value gradually decreases from 0.84 to 0.72, and the *FWHM* drops almost linearly from 144 to 23 nm. So, we theoretically obtain a large bandwidth tunability of graphene absorption enhancement by changing the period *p*. The bandwidth tunability is closely related with the variation in the coupling strength of delocalized SPPs and localized MPs. The SPPs are highly confined on the surface of the silver substrate, and their electromagnetic fields exponentially decay into the air. So, the SPPs usually have a low radiative damping [58]. In addition, the imaginary part of the silver permittivity is small in the near-infrared region [50], and thus the SPPs also have a low Ohmic loss. Because of the low radiative damping and Ohmic loss, the lifetime of the SPPs is very long. This is the reason why the *FWHM* is remarkably small.

The quality factor *Q*, the dephasing time *T*_*d*_, the effective mode volume *V*_*eff*_, and the Purcell factor *F* are also the critical components in high-*Q* plasmonic systems. These physical quantities are defined as the following [59–61]:

3 $$Q = {{\lambda_{res} } \mathord{\left/ {\vphantom {{\lambda_{res} } {FWHM}}} \right. \kern-0pt} {FWHM}}$$

4 $$T_{d} = {{2\hbar } \mathord{\left/ {\vphantom {{2\hbar } \Gamma }} \right. \kern-0pt} \Gamma }$$

5 $$V_{eff} = \iiint {\frac{{\varepsilon \left| E \right|^{2} }}{{Max(\varepsilon \left| E \right|^{2} )}}}dV$$

6 $$F = \frac{3Q}{{4\pi^{2} V_{eff} }}\left( {\frac{{\lambda_{res} }}{n}} \right)^{3}$$

For example, we have calculated the above physical quantities at two absorption peaks with a period of 800 nm. The resonance wavelength λ_res_ of the absorption peaks is 801.2 and 1239.7 nm, and the *FWHM* is 1.28 and 144.6 nm. The calculated *Q* is 625.9 and 8.57, respectively. The linewidth* Γ* of the absorption peaks is 2.49 and 107.47 meV, and the calculated *T*_*d*_ is 528.9 and 12.3 fs, respectively. By numerical calculations, the *V*_*eff*_ is 5.69*10^6^ and 4.05*10^5^ nm^3^, and the corresponding *F* is 4284 and 3066, respectively. In our studied structures, the above physical quantities are comparable with the obtained values in some common plasmonic systems [59–63]. Recent studies have shown that the high-*F* can enhance the quantum yield from carbon nanotube excitons coupled to plasmonic nanocavities, and can also enhance the photoluminescence emission from hot carriers in gold nanorods [64–66]. In addition, the plasmonic density of states (*PDOS*) is also a critical component in plasmonic systems [65, 66]. A plasmon resonance cavity can enhance the *PDOS* by a Purcell factor *F* [59], that is, the *F* is proportional to the *PDOS* [66].

**Fig. 4:**
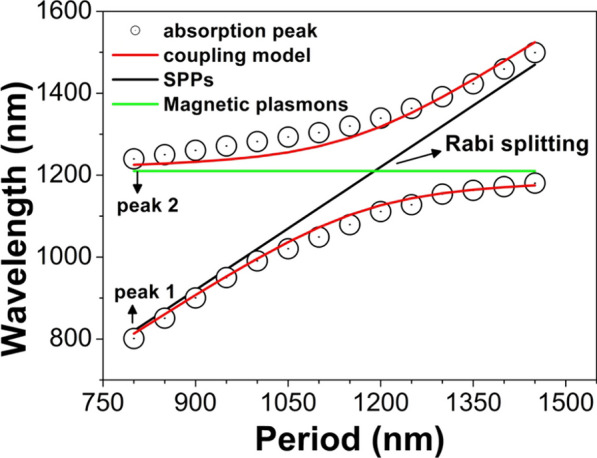
Resonance wavelength for different period

The physical mechanism of the above absorption peaks can be revealed by using a coupling model of double oscillators to estimate the peak positions of different periods, as shown in Fig. 4. The green line gives the position of MPs, whose resonance wavelength is *λ*_*MP*_ = 1210 nm and corresponds to a photon energy *E*_*MP*_ = 1.025 eV. The MPs are highly localized into the slit, whose resonance wavelength is mainly determined by the width and the depth of the slit but is almost independent on the period *p* of the slit array. The black line shows the positions of SPPs for different periods, and the resonance wavelengths of SPPs are calculated by a formula [58]:

7 $$\lambda_{spp} = {p \mathord{\left/ {\vphantom {p {\sqrt {{{\varepsilon_{Ag} } \mathord{\left/ {\vphantom {{\varepsilon_{Ag} } {(\varepsilon_{Ag} + 1)}}} \right. \kern-0pt} {(\varepsilon_{Ag} + 1)}}} }}} \right. \kern-0pt} {\sqrt {{{\varepsilon_{Ag} } \mathord{\left/ {\vphantom {{\varepsilon_{Ag} } {(\varepsilon_{Ag} + 1)}}} \right. \kern-0pt} {(\varepsilon_{Ag} + 1)}}} }}$$

where *ε*_*Ag*_ of the relative permittivity of silver substrate. The corresponding photon energy *E*_*SPP*_ for SPPs to be excited can be also calculated according to the above formula. It is well-known that SPPs are delocalized and propagate on the surface of silver substrate, so their resonance wavelengths are relevant to the period *p*. The hybridization of delocalized SPPs and localized MPs forms two hybridized modes [46, 67], and the excitation energies of hybridized modes are:

8 $$E_{ + , - } = {{(E_{{{\text{MP}}}} { + }E_{{{\text{SPP}}}} )} \mathord{\left/ {\vphantom {{(E_{{{\text{MP}}}} { + }E_{{{\text{SPP}}}} )} 2}} \right. \kern-0pt} 2} \pm \sqrt {g^{2} { + }{{(E_{{{\text{MP}}}} - E_{{{\text{SPP}}}} )^{2} } \mathord{\left/ {\vphantom {{(E_{{{\text{MP}}}} - E_{{{\text{SPP}}}} )^{2} } 4}} \right. \kern-0pt} 4}}$$

where *g* is the hybridization strength. At the energy cross between MPs and SPPs, the similar phenomenon of Rabi splitting appears, as indicated by the black arrow. The Rabi splitting energy is equal to 2* g*. By taking *g* = 80 meV, we can well predict the peak positions of the graphene absorption. It is obvious that two red lines obtained from the above model have a good fit with the peak positions (black circles) for different periods.

**Fig. 5:**
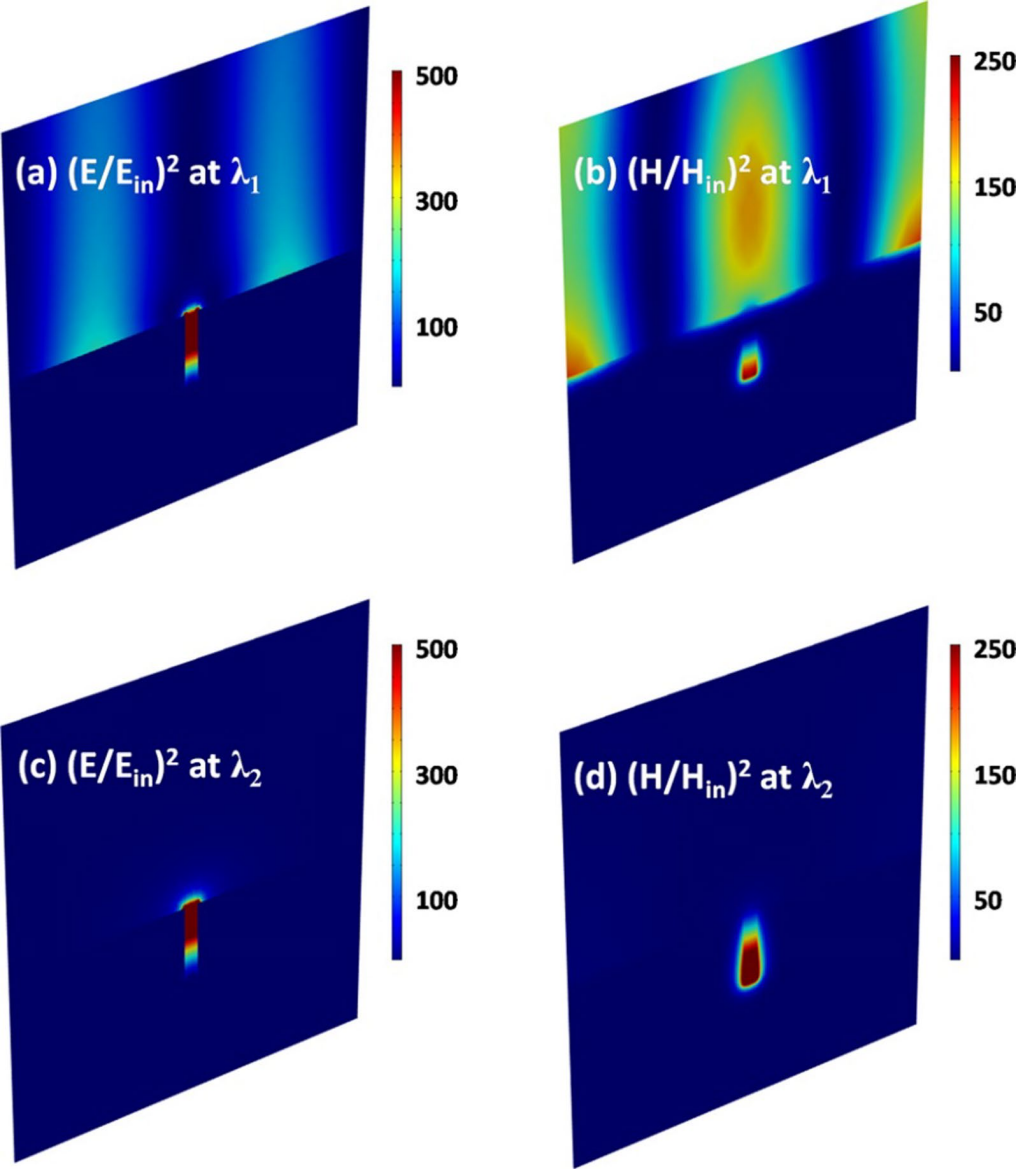
Electromagnetic field distribution at two resonance wavelengths

To further explain physical mechanism of the graphene absorption peaks, in Fig. 5 we plot the electric and magnetic fields on the *xz* plane for two resonance wavelengths (*λ*_*1*_ = 801.2 nm and *λ*_*2*_ = 1239.7 nm) of peak 1 and peak 2. It is clearly seen in Fig. 5c, d that at *λ*_*2*_ the electric fields are mainly distributed near the mouth of the slit, and the magnetic fields are highly confined within the bottom of the slit. This kind of field distribution directly indicates the excitation of localized MPs [68, 69]. At *λ*_*1*_, the noticeable electric and magnetic fields appear alternately on the surface of silver substrate in Fig. 5a, b. Such a field distribution suggests that delocalized SPPs are excited efficiently [58].

**Fig. 6:**
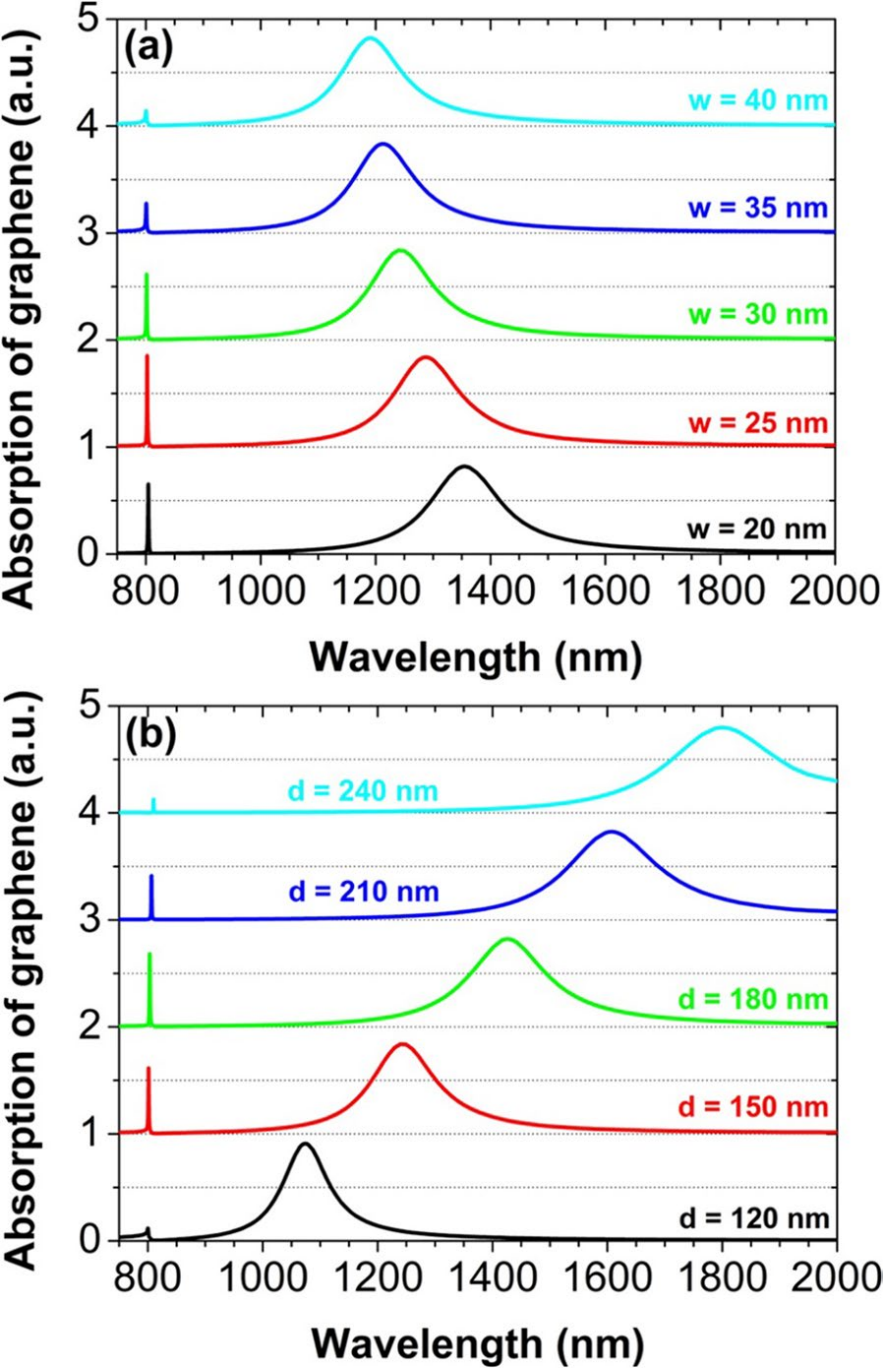
Graphene absorption spectra for different slit width *w* (**a**) and depth *d* (**b**). The period is 800 nm

In Fig. 6, we have also investigated in detail the effect of the geometry size of the slit on the absorption of graphene. When the width *w* or the depth *d* of the slit is changed, the narrow-band absorption peak does not shift almost. The physical reason is that the peak is mainly related to the delocalized SPPs whose resonance wavelength is unchanged for a fixed period, even if the slit size is changed. However, the excitation strength of SPPs suffers a change, so the maximum absorption at the peak is different. In contrast, the broad-band absorption peak has an obvious blue or red shift, for the width *w* or the depth *d* to be increased, respectively. This is because the peak is closely relevant with the localized MPs whose resonance wavelength is determined by the geometry size of the slit [68]. But, the maximum absorption at the peak has no obvious change, due to the relatively stable excitation strength of MPs.

**Fig. 7:**
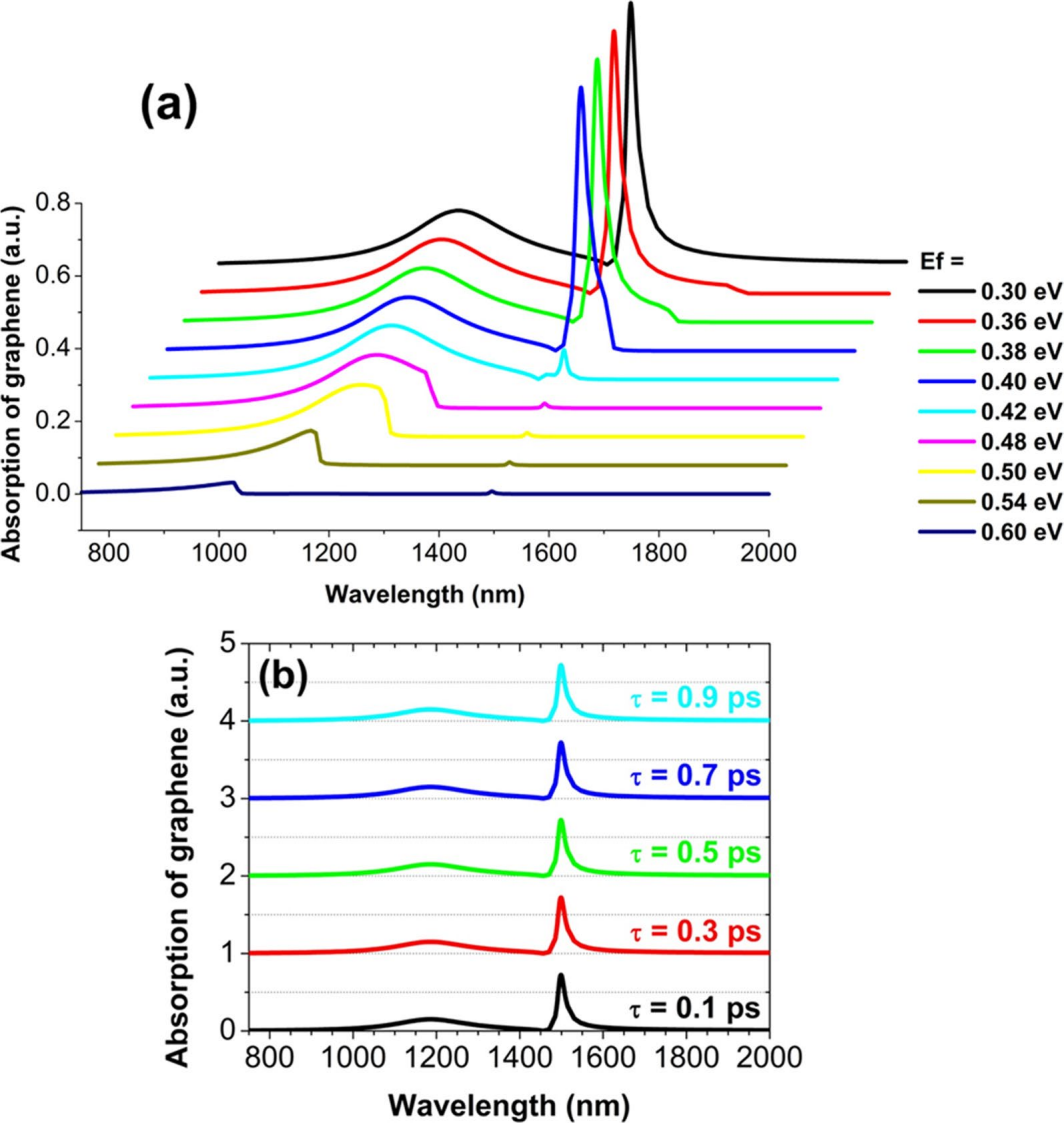
Graphene absorption spectra for different Fermi energy *E*_*f*_ (**a**) and relaxation time *τ* (**b**). The period is 1450 nm

Finally, we explored the impact of Fermi energy *E*_*f*_ and relaxation time *τ* on the absorption of graphene. When *E*_*f*_ is increased from 0.30 to 0.40 eV, the right sharp absorption peak has no obvious change, as shown in Fig. 7a. However, for *E*_*f*_ to be increased to 0.42 eV, the peak value drops abruptly from 0.72 to 0.07. With *E*_*f*_ further increased, the graphene absorption can further drop to almost zero, because in this case the imaginary part of the graphene permittivity becomes very small. So, by changing *E*_*f*_, we can modulate the graphene absorption from a maximum to almost zero with a nearly 100% modulation depth, and thus achieve a switch effect which has a potential application in light wave modulators [7, 70, 71]. The left broad absorption peak exhibits a similar change trend when *E*_*f*_ is increased continually from 0.30 to 0.60 eV. It is clearly seen in Fig. 7b that two absorption peaks almost have no change, for the relaxation time *τ* have a large increase from 0.1 to 0.9 ps. The reason is that the graphene permittivity changes only slightly for different τ in the investigated wavelength range from 750 to 2000 nm.

## Conclusion

We theoretically studied the bandwidth tunability of the graphene absorption enhancement in the near-infrared wavelengths. The monolayer graphene was placed on the silver substrate surface with a periodic slit array. Two graphene absorption peaks were observed to result from the hybridization of delocalized SPPs and localized MPs. The peak positions could be predicted by a coupling model of double oscillators. The absorption bandwidth (*FWHM*) was largely tuned from about 1–200 nm, by changing the period of the slit array. The effect of the geometry size of the slit on the absorption of graphene was also investigated in detail. Our work is promising in applications for photoelectric devices.

## Data Availability

The data and materials are available of this article.
